# UK Infected Blood Inquiry–An Historical Appraisal

**DOI:** 10.1111/hae.70307

**Published:** 2026-05-17

**Authors:** Christopher A. Ludlam

**Affiliations:** ^1^ Centre For Cardiovascular Sciences School of Neurological and Cardiovascular Sciences, University of Edinburgh Edinburgh Midlothian UK

**Keywords:** AIDS, blood safety, haemophilia, HIV, no fault compensation, public inquiry

## Abstract

**Introduction:**

This appraisal considers the 2018 UK Infected Blood Inquiry and its Report (2024) in the context of the history of haemophilia treatment developments over the past fifty‐five years, and the tragic impacts of the AIDS pandemic.

**Aim:**

The paper appraises the conduct and findings of the IBI and its Report in relation to HIV and AIDS.

**Method:**

This appraisal represents a personal perspective informed by the author's experience from 1969 of treating people with haemophilia and allied blood disorders, and by contemporaneous scientific publications.

**Results:**

The Report is commended for recommending compensation for infected patients and their families and for recognising developments that enhanced blood safety in the past 50 years. Nevertheless, this appraisal observes that a UK government‐backed system of no fault compensation is long‐overdue. Failure in the late 1980s to offer one for HIV has prolonged and accentuated the trauma of AIDS for all involved. This perspective hypothesises that in the circumstances under which the IBI was commissioned any belated compensatory award depended on finding culpable responsibility in haemophilia treatment services. The Report's significant omissions, oversights, and judgements with hindsight, were thus deemed necessary to support recommendations for government offers of compensation.

**Conclusions:**

The Report disappoints in not delivering to patients and their families a more complete, evidence‐based history of HIV infection in the context of haemophilia and its treatment. The Report thereby omits acknowledgement of the dedication and professionalism with which members of treatment services collaborated to combat the threats of blood‐borne infections.

## 1 Introduction

The author appraises aspects of AIDS and HIV considered by the UK Infected Blood Inquiry (IBI) and its Report (hereafter the ‘Report’) [1]. This assessment, following the maxim, ‘context is everything’, critiques the lack of contextual frameworks under the following headings:

2 Historical context
3 The tragic appearance of AIDS
4 The Emergence of HTLVIII as the cause of AIDS in Haemophilia
5 Interpretation of an Anti‐HTLVIII Assay Result
6 UK governmental response to AIDS
7 The IBI process
8 Overview of the Report
9 Highlighting specific omissions
  i Clinical description of haemophilia
  ii Epidemiology and emergence of AIDS and HIV and implications for evidence‐based treatment choices in mid‐1983; cryoprecipitate versus concentrate
  iii Early uncertainties about causes and diagnosis of AIDS
  iv Comparison with responses to AIDS elsewhere in Europe
  v The matter of ‘paramountcy’ of ‘patient safety’
10 Edinburgh Haemophilia Centre—a case example

## 2 Historical Context

Since 1969, the author has treated patients with haemophilia and associated conditions. In 1980, he became director of the Haemophilia and Thrombosis Centre in Edinburgh, serving the population of south‐east Scotland. During his professional life‐time experience of developing services for heritable bleeding disorders within the UK and internationally, he grappled with the many initial issues raised by the appearance of AIDS in the early 1980s, with non‐A non‐B hepatitis (NANBH), other viral infections, and variant CJD (vCJD).

Haemophilia, a predominantly inherited condition, affects patients and their families from cradle to grave. Alongside them, dedicated services have developed to offer life‐long treatment, forging close relationships. As is well known, without treatment, haemophilia results in crippling arthritis and a shortened lifespan, with many dying as teenagers or young adults from intracranial or gastrointestinal haemorrhage [2]. Over the past 50 years, developments in therapy have made it possible to grow up with normal, or near normal, joints and almost normal life expectancy [3, 4]. Since the 1960s, patients and physicians have appreciated that these welcome benefits of therapy must be continually weighed against newly perceived risks of therapy. While, fresh frozen plasma and cryoprecipitate offered only modest improvements in life expectancy, use of concentrates, especially if patient self‐administered at home and prophylactically, resulted in life expectancy approaching that of the rest of the population [5]. The lives of both children and adults were consequently transformed by regular attendance at school and work [6]. Until the early 1980s and the arrival of AIDS, it was universally agreed that the benefits of concentrates outweighed both their infective and inhibitor risks, although reviewing the balance between benefits and risks continually challenged physicians and patients [7].

Fortuitously, the Covid 19 virus and genome was characterised within weeks of that new illness being recognised in late 2019, enabling its spread in the pandemic in 2020 to be quantified, and an effective vaccine developed within a year [8]. By comparison, there were nearly 30 years’ uncertainty during which manifestations of ubiquitous and unavoidable NANBH had to be managed before a viral marker was characterised in 1989 [9]; 3 years of similar difficulty occurred before HTLVIII was identified as the cause of AIDS in 1984 [10]. Relatively early in the 1970s, it became clear that only 10–20 cryoprecipitate infusions were sufficient to cause NANBH, whereas the extent of HIV infection only emerged in 1985.

Overall, the Report's retrospectively judged a‐historical account of haemophilia treatment in the last fifty years does a disservice to all those infected and affected family members [7]. They have suffered considerably and deserve an accurate understanding of the tragic circumstances they endured.

3

The early history of AIDS in haemophilia is seared into the memories of those who lived through the 1980s because of the stress and uncertainty of dealing with the many immediate difficulties that arose. The following historical account summarises what made the impact of this pandemic on haemophilia such a devastating and complex clinical challenge.

The reporting of pneumocystis pneumonia and Kaposi's sarcoma in young men in New York and California in 1981 heralded a new disease of unknown cause until its viral aetiology was published in May 1984 [10]. Prior to this date, diagnosis was purely clinical and because those identified had clinical evidence of severe immune depression, it was named Acquired Immune Deficiency Syndrome (AIDS). Its relation to the clinical constellation of features that became known as ‘Pre‐AIDS’ and ‘AIDS related Complex’ was much debated. The diagnostic difficulty was compounded by serial changes in the clinical criteria defining AIDS and these possibly associated conditions [11]. By 1982, AIDS cases had been identified in four groups known as the 4Hs—homosexuals, haemophiliacs, Haitians and heroin users [11]. The situation was further confused because the clinical presentation was different between these groups. For example, Kaposi's sarcoma was only reported in gay men (now known as due to a different virus Human Herpes Virus 8 [12], which is only transmitted by intact viable cells that are destroyed when blood donor plasma is frozen prior to fractionation into clotting factor concentrates). Evidence accumulated to support the hypothesis that if AIDS was a viral infection it appeared to have a long ill‐defined incubation period. Once the causative virus was identified as HTLVIII (later known as HIV) [10] and an anti‐HTLVIII test developed to detect exposure to the virus, testing of stored blood samples revealed that the first infections had occurred in those with haemophilia in 1978 [13, 14] in the USA and 1979 in the UK. Thus, in the following three to five years there were few symptoms, while patients continued to be infected by concentrates prepared from asymptomatic donors in whom the infection remained unidentified.

## 4 Appearance of AIDS in Haemophilia

In July 1982, three patients with haemophilia A and pneumocystis pneumonia were reported in the USA [15]. At the end of 1982, a further 8 had been identified in the USA, and by the end of 1983, there were an additional 14 cases out of approximately 20,000 individuals with haemophilia [16]. In autumn 1982, United Kingdom Haemophilia Directors Organisation (UKHCDO), under the conscientious chairing of Professor Arthur Bloom, introduced measures to respond to the evolving situation and collaborate with others internationally. It was then predicted in the UK that by the end of 1984, there might be between two and four AIDS cases. Subsequently, this prediction proved to be accurate as there were two cases in 1983 and one in 1984. With this small number of expected diagnoses out of about 2500 patients treated annually, it was not easy to judge what changes to therapy would be appropriate, bearing in mind treatment's life‐saving potential. UKHCDO's recommendations mirrored those being implemented in the USA's National Haemophilia Foundation [17]. Without any diagnostic blood test for AIDS, the only possible ‘surrogate’ marker was to assess cell‐based immunity by measuring T helper (reduced) and T suppressor (normal or increased) with the Th/Ts ratio reduced. Several investigations of apparently well, asymptomatic patients with haemophilia were quickly conducted in the USA. These revealed that a large proportion with severe haemophilia had a reduced Th/Ts ratio [18], raising the possibility of a wide‐spread viral infection in people with haemophilia. Another possibility was that these immune changes were due to immune modulating effects of therapeutic concentrates, as they contained considerable quantities of immunoglobulin as well as other proteins, an effect quite separate from a potential virus [19, 20]. In response to these reports, many Haemophilia Centres worldwide began monitoring Th and Ts cell numbers in peripheral blood samples taken at routine review clinics.

## 5 Emergence of HTLVIII Virus as the Cause of AIDS in Haemophilia

Speculation about the cause of AIDS began immediately after the first cases were reported and continued into the spring of 1984. Even in February 1984, just months before HTLVIII was reported to be the cause, the eminent blood‐safety virologist, Professor Richard Tedder, reported at the National Institute for Biological Standards and Control meeting on the ‘Infective Hazards of Blood Products’ that the cause of AIDS in haemophilia lay between an ‘infective agent’ and ‘overwhelming of the immune system by repeated infusion of foreign proteins’ [21]. As summarised above, speculation ended in May 1984 with the identification of HTLVIII. What became even clearer after the causative virus was identified was that those patients with haemophilia who were anti‐HTLVIII negative had Ts and Th cell aberrations due to non‐viral components of concentrates (probably immunoglobulin [22]). Moreover, subsequent studies provided evidence that these immune changes were associated with an increased risk of infection when exposed to HTLVIII [23, 24].

## 6 Interpretation of an Anti‐HTLVIII Assay Result

After the initial anti‐HTLVIII test was developed, there were uncertainties about the accuracy and interpretation of a positive as well as a negative result [25, 26]. The initial research assays associated with false positive and negative results persisted until at least May 1985 in the UK [27]. Subsequently, improved methodology increased their accuracy. Early reports indicated high levels of positivity, especially in recipients of USA commercial concentrates, but as the majority were clinically well, it was initially unclear whether patients who were anti‐HTLVIII positive had only been exposed and cleared the virus, or whether they were actively infected. Detection of HTLVIII virus had to await the development of an ELISA assay in 1985, revealing that the majority of anti‐HTLVIII positive patients were actively infected with the virus [26].

**FIGURE 1:**
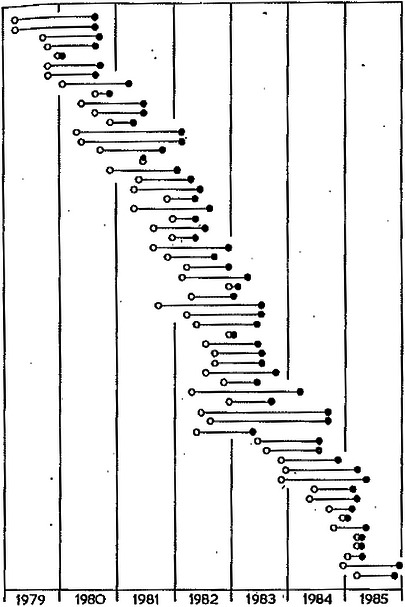
Estimated dates of anti‐HIV seroconversion in 59 patients with haemophilia are midpoint between last negative and first positive result. Open and closed circles denote anti‐HIV negative and positive results respectively. Lee et al. [14].

**FIGURE 2:**
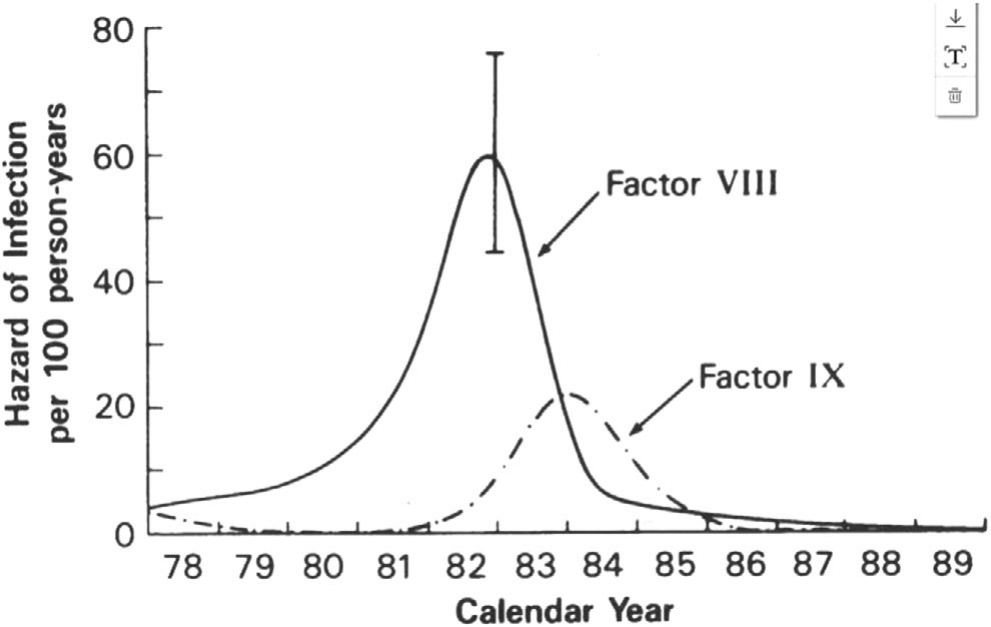
Estimate of the HIV hazard rate for USA patients with haemophilia who used factor VIII or IX concentrate (Error bar shows the 95% confidence interval for factor VIII at January 1983. Goedert et al. [28].

With subsequent retrospective testing of stored blood samples, it became clear in 1989 that by mid‐1982, when clinical AIDS was first reported in haemophilia, half of those later diagnosed as infected had already been infected, both in the UK and USA, and by mid‐1983 this had risen to three‐quarters (Figures 1 and 2) [13, 14, 28]. The crucial significance of this is highlighted below (see 6ii) and emphasised by Lee [7] and Farrugia [29, 30].

HIV infection in haemophilia paralleled exposure to factor VIII (and to a much lesser extent factor IX) concentrate use across the world. Within Western Europe, countries reliant on commercial USA concentrates had high and similar rates of anti‐HTLVIII positivity rates and subsequent AIDS cases.

## 7 UK Governmental Response to AIDS

Regrettably, by the late 1980s, the devastation by HIV to individuals and their families was not yet appreciated by the UK government, which declined to offer no fault compensation. In 1989, against advice and advocacy from 1987 by physicians, including this author, and others, the Minister of Health decided that to obtain compensation patients should prove negligence by blood transfusion services, physicians, and health authorities. This decision initiated a trail of distress for over thirty years for all involved, as it set the patient group against their treating physicians to seek redress for what had been an unpredicted existential disaster. Because haemophilia is a rare disorder, in which relatively few specialist physicians (then approximately one for each major UK city) had the appropriate expertise, patients had no alternative source of treatment.

Over the past 30 years, governments have established financial support schemes, but they are considered inadequate. The non‐governmental 2007 Archer Inquiry report recommended the provision of fair compensation [31]. In 2008, the Scottish government established a Public Inquiry to review HIV and HCV infection. Its terms of reference specifically excluded consideration of compensation, which Scotland's Health Secretary, Nicola Sturgeon, promised would be decided when the inquiry reported. The resultant Penrose Report (2015) [32], following a very thorough, evidence‐based examination, concluded that it ‘revealed few respects in which matters should, or more importantly could, have been handled differently’ [33]. This failed to satisfy those infected by these viruses and their affected families.

In 2015, David Cameron, then UK Prime Minister, ‘apologised on behalf of the government to victims of the contaminated blood scandal’ [34]. Later in 2018, Prime Minister, Teresa May, stated that, ‘The contaminated blood scandal of the 1970s and 80s is an appalling tragedy which should simply never have happened’ [35]. It is unclear whether ‘appalling tragedy’ referred to the infections, or the government's response. It is clear that by this time the government's view reflected public appreciation of the devastation caused to patients and their families. It was disappointing, however, that it did not feel empowered, belatedly, to arrange compensation. Instead, it established the UK‐wide IBI, apparently anticipating that a finding of negligence by providers of services would enable the government to offer compensation. It seems to this author, therefore, that the IBI had little autonomy to deviate from endorsing a presupposed ‘scandal’ before it had even started collecting evidence. If this led to the adoption of a presumption of guilt on the part of the treating services, it was at the expense of traducing their integrity and professionalism, and of jeopardising their on‐going caring relationships with the patient community.

This Journal has recently published an important contemporaneous clinical expert's view of the Report [7], and the reasoned critical views of three eminent plasma fractionators (one of whom is also a patient) [29, 36, 37]. An additional critique offers a further broader international perspective of inquiries into blood safety, and has made pertinent observations of the IBI [30].

## 8 The IBI Process

The IBI's very broad remit was to review infections by blood and blood products, including HIV, HCV and vCJD, in recipients of blood transfusions and those with heritable bleeding disorders. Like the Penrose Inquiry, infected and affected family members were encouraged to submit Witness Statements; many provided oral evidence in person at public sessions.

Unfortunately, the IBI's process of information retrieval and sharing was limited. Treating physicians could respond in writing to criticisms of clinical management made throughout the evidential hearings, for eventual publication on the Inquiry website. Many, however, were denied ready access to contemporaneous medical records, even when these were available. This also disadvantaged patients by preventing them from receiving additional information to supplement their memory and understanding of events from decades earlier.

When the Inquiry opened, more than fifty years had passed since some examined events had occurred. Many who were infected, or had provided care, were too frail, ill, or had died, and were therefore unable to testify at all [7, 37]. Those involved, whether directly or indirectly, in treatment services were required to submit Witness Statements in response to the Inquiry's extensive detailed questions. Witness Statements from NHS staff were generally not presented in entirety at the Inquiry's open public sessions, with the witness invited to amplify and justify their content by examination undertaken by Counsel to the Inquiry. Whereas a Public Inquiry is intended to be an inquisitorial process, with the oral examination serving to tease out details in witnesses’ evidence to clarify and test its veracity, IBI witnesses were often presented with historical documents from many decades earlier and invited to comment immediately on their content. Many found the questioning abrupt, confrontational, and adversarial.

Written Witness Statements by some physicians, not invited to provide oral evidence, were not drawn to the public's attention other than being made available on the website. There was neither an index for these, nor does it appear their evidence was appropriately identified in the Report. As a result, some important evidence was effectively ignored.

## 9 Overview of the Report

The Report is to be commended for making the welcome, and politically useful, recommendation that financial compensation for blood‐borne HIV infections should be given to those infected and their affected families. This was not, however, no fault compensation. Rather, the Report finds ‘people have been failed, not once but repeatedly, by their doctors, by the bodies (NHS and others) responsible for the safety of their treatment, and by their governments’ [38]. Clinical services are criticised for many of their attempts to address the evolving clinical uncertainties about HIV, with little acknowledgment of the difficulties involved, or of the response by UKHCDO to the potential impacts that these challenges had on service provision. Minimal consideration, or credit, was given to the much later threat of, and response to, vCJD in the mid‐1990s [39, 40].

The IBI's recommendations to enhance blood safety approved many measures already established in the intervening thirty years, including UKHCDO's promotion of patient safety through its extensive patient National Haemophilia Database (NHD) and its thirty‐year audit of haemophilia centres. Preferential use of recombinant concentrates was also recommended by UKHCDO following their licensing in mid‐1990s, although implementation was delayed for several years due to lack of government funding.

Overall, the report disappoints through its lack of balance, its incomplete history of events, and its understanding of the challenges posed by infective agents. Nevertheless, the report (Vol. 4 footnote 1681) does publish, in small print, this author's response to the IBI Draft Report, as doing ‘a gross disservice to a group of patients and their families who have already suffered considerably and waited too long for their distress to be recognised by the state. What survivors of those with heritable bleeding disorders and their families seek is a balanced and reasoned assessment of the history of this traumatic and challenging period.’

Contrary to the convention of judging past events and practice by contemporaneous standards, rather than with the benefit of hindsight, the Report predominantly bases its assessments on current knowledge and practices. With some notable exceptions, its expert panels had little personal experience of medical practice during the period under review. Assertions of past negligence significantly contributing to infections ought to be substantiated by statements about practices and procedures then prevailing. The Inquiry, despite encouragement, failed to seek independent assessment of UK provision of haemophilia services from external or non‐UK expert physicians. By contrast, the Penrose Inquiry Chair had continual access to a Medical Assessor, Professor Oliver James, Emeritus Professor of Medicine, University of Newcastle, to clarify medical matters.

These observations raise questions as to standards in conducting a Public Inquiry. Criticisms have been set out in two UK Parliamentary House of Lords Reports (2014, 2025) [41]. There, Lord Howe, emphasises the importance of ‘establishing the facts—providing a full and fair account of what happened, especially in circumstances where the facts are disputed or the course and causation of events is not clear’. The government has given a commitment to review and reform Public Inquiry processes; perhaps experiences from the IBI will feed into this. Moreover, Lord Hodge, deputy president of the UK Supreme Court, urged the legal profession to improve their ‘scientific and technical literacy to prevent miscarriages of justice’ [42].

## 10 Highlighting specific Omissions

This assessment of the report does not aim to be comprehensive but highlights five significant omissions in the consideration of data and misinterpretation of evidence concerning haemophilia and its treatment.

i. **Clinical Description of Haemophilia and Its Treatment**: The IBI failed to appreciate haemophilia related morbidity and reduced life‐expectancy. The Report concluded with what it perceived to be the risks of AIDS and HTLVIII from plasma‐derived blood products and what action should have been taken in response, without any consideration of benefits – or, indeed, the necessity of therapy. The practise of medicine requires that benefits are always weighed against risks; not to do so seriously undermines the Report's recommendations.
ii. **Epidemiology and Emergence of AIDS and HIV and Implications for Evidence‐based Treatment Choices in mid‐1983; Cryoprecipitate versus Concentrate**: The Report omits consideration of the extensive published epidemiology of AIDS and HIV in the UK, stating that decisions should have been made in mid‐1983 to halt imports of commercial concentrates and offer cryoprecipitate instead. A high‐level broad‐based group of relevant national experts had considered this in July 1983 in the light of the evidence. It ‘concluded that this was not feasible in the UK on grounds of supply’ [43]. The IBI's proposal and its apparently unforeseen consequences has already been explored by three plasma fractionators in this Journal [29, 36, 37].

As evidenced from the history of AIDS set out above, we now know that by mid‐1983, at least three quarters of those with haemophilia who would be later diagnosed with HIV were already infected (Figure 1) [14, 44]. The Report chooses not to acknowledge this inconvenient truth. Would this recommendation have met the General Medical Council's requirement to ‘propose, provide or prescribe effective treatment based on the best available evidence’ [45]? The Inquiry declined to accept a supplementary Witness Statement from this author, reporting that concentrate prepared from a low‐AIDS prevalence population (similar to the UK) could be safer than cryoprecipitate as had been observed in Sweden [46].

Having made its proposal to prefer cryoprecipitate, it would have been more informative had the Report assessed the potential benefits and risks of this recommendation, as reflected in Good Medical Practice [45]. Without such a reasoned evidence‐based assessment of their consequences, some IBI recommendations appear non‐scientific, simplistic, and naïve.

iii. **Early Uncertainties About Causes and Changing Diagnostic Criteria for AIDS, ‘pre‐AIDS’, and ‘AIDS Related Complex’, and Limitations of Early Anti‐HTLVIII Testing**: The Inquiry had difficulty in appreciating the uncertainties arising from changing clinical criteria in diagnosing AIDS, ‘Pre‐AIDS’, and ‘Aids related Complex’ in the early 1980s as described above. Even had its viral aetiology been assumed from the beginning, its transmissibility and the extent to which AIDS would affect UK individuals with bleeding disorders were impossible to predict.

The IBI was also reluctant to acknowledge the uncertainties about interpreting the initial anti‐HTLVIII [25]. Even by May 1985, after nine months’ experience of UK anti‐HTLV‐III testing, these uncertainties were clearly expressed in the Chief Medical Officer for England's letter to all doctors [27]. The IBI appears not to have understood the ethical difficulties of giving such ambiguous results to all patients, whether positive or negative.

iv. **Comparison Between AIDS Appearance and Management in the UK and Other Countries**: The Report is almost silent about how AIDS affected haemophilia and its treatment in other countries. Inclusion of this would have shown that the UK's experience and the treatment response was very similar to that in other comparable Western European countries.
v. **The Matter of ‘Patient Safety’ as ‘Paramount’**: When presenting his Report, the Chair stated: ‘Many, indeed most, infections would have been prevented if patient safety had been paramount throughout’ [38]. Had physicians viewed safety as ‘paramount’, infections, such as NANBH and HTLVIII, could only have been prevented between 1960 and 1990 by withholding all plasma‐derived factor VIII or IX therapy for joint or muscle bleeds, and potentially life‐threatening intracranial or gastrointestinal haemorrhages. As all potential new treatments carry possible risks, a philosophy of patient safety as ‘paramount’ would stifle virtually all therapeutic medical research, including trials to develop improved treatments for a wide range of clinical conditions.

## 11 The Edinburgh Haemophilia Centre—A Case Example of Developing a Wide Spectrum of Clinical Care Services

The report appraises the larger UK haemophilia centres. Unfortunately, many of their directors from the 1980s were not available, because of illness or death, to inform the Inquiry about their responses to the appearance of AIDS. This author provided extensive written and oral evidence about the Edinburgh Haemophilia Centre [25], much of which the Report ignored, misreported, or misinterpreted. Doubts may thus arise regarding its assessment of other UK centres. Interested readers may draw their own conclusions from reading its account: [1] (Vol. 4 pp 296–300). Extensive evidence was also submitted to and reported in the Penrose Inquiry: [32] (PEN.012.0386, PEN.015.0376, and PEN.012.0353) and made available to the IBI.

In Edinburgh, unlike most other centres, patients were treated solely with blood products produced from local blood donors by Scottish National Blood Transfusion Service (SNBTS). Avoiding commercial concentrates reduced risks of introducing non‐native viruses. Combined with increased surveillance to keep abreast of continual challenges to blood safety in the early 1980s, this policy provided the first evidence in autumn 1984 that the NHS blood supply had infected patients with HTLVIII. A batch of NHS factor VIII concentrate transfused to patients in the spring of that year infected 15 of 33 recipients at a time when there were no reported AIDS cases in the general population in Scotland [24]. Prior to this observation, it was assumed, and assessed by SNBTS, that the risk from apparently healthy volunteer blood donors of a putative AIDS virus was very small [21]. These issues were thus appropriately of major interest to the IBI.

In assessing the Centre's activities, however, its Report misinterprets data it quotes and makes inaccurate criticisms and speculations about arrangements at the Centre, particularly with reference to the work of the author as the new director in the early 1980s. This section of the Report disappoints in missing the opportunity to present significant medical developments with ‘fairness’, as directed by the UK Parliament's 2005 Inquiries Act.

The Report shows a questionable understanding of how patient monitoring should adapt to changing knowledge and threats, and a puzzling confusion between ‘monitoring’ and ‘research’, as supported elsewhere [7]. In 1983, lymphocyte subset testing was introduced to evaluate patient wellbeing in response to abnormalities being detected in USA patients. The results were published following an inquiry in a leading medical journal [19, 20]. The Report, in error, concludes that, as it was published, the tests constituted ‘research’. The Report has difficulty in differentiating between ‘evaluation’, ‘research’ and ‘audit’, even as set out in the current 2025 UK guidance by the government's Policy Framework for Health and Social Care Research (HSCR). The Report's reasoning reveals a fundamental misconception of the clinical assessment of patients and reporting of the results of investigations. Furthermore, it appears not to appreciate the distinction between ‘invasive’ explicit research investigations, for which patient consent was obtained, and ‘non‐invasive’ monitoring investigations. As noted above, access to an independent medical assessor might have spared the IBI such misperceptions.

High‐quality clinical services must evolve to incorporate advances in therapy and address any threats these pose. The adaptation of the Edinburgh Centre's provision of care to the developing therapeutic landscape and its difficulties has been referenced above. Its clinical quality was assessed triennially in the UK national clinical audit; patients’ views were invited by anonymous questionnaires, and the Centre reviewed by a detailed protocol‐driven, systematic process by an external haemophilia physician, nurse, and patient [47]. All detailed reports were available to the IBI but are not referenced in the Report.

## 12 Conclusions

Blood and blood products have been lifesaving for countless individuals. The terrible tragedies of infections they transmitted have too long haunted patients in the UK because of a government decision to distance itself and to refuse, in the late 1980s, to acknowledge the devastating consequences of HIV. A more compassionate and responsible reception by the Department of Health, as happened in many other countries, would have spared patients, their families, and health service care providers much subsequent distress (as well as considerable governmental legal costs) over the ensuing decades.

The Report gives the impression of managing the complexity of its task by offering untested simplistic criticisms while overlooking the consequences of some of its controversial proposals. It displays a notable disregard of scientific and historical evidence. When the Inquiry was established in 2018, the view appeared to be that after many years of neglect, compensation was due. Belatedly, therefore, the government commissioned the IBI, hoping that its Report, after a further delay of 6 years, would conclude that those who had committed themselves professionally and personally to provide the service were negligent. The resulting long‐term consequences for the NHS and the standing of the legal profession and politicians are only now beginning to be appreciated.

Patients, their families, NHS staff, and importantly the wider public, expect a Public Inquiry to deliver an accurate summary of the events and with evidence‐based conclusions. The Report constitutes a missed opportunity to deliver this and to bring real closure to all affected.

## Author Contributions

Written by Christopher A. Ludlam.

## Funding

The author has nothing to report.

## Conflicts of Interest

The author declares no conflicts of interest.

## Data Availability

Data sharing is not applicable to this article as no new data were created or analysed in this study.
